# Gene Expression Alterations in Immune System Pathways in the Thymus after Exposure to Immunosuppressive Chemicals

**DOI:** 10.1289/ehp.1002358

**Published:** 2010-11-01

**Authors:** Rachel Frawley, Kimber White, Ronnetta Brown, Deborah Musgrove, Nigel Walker, Dori Germolec

**Affiliations:** 1 National Institute of Environmental Health Sciences, National Toxicology Program, Research Triangle Park, North Carolina, USA; 2 Department of Pharmacology and Toxicology, Virginia Commonwealth University, Richmond, Virginia, USA

**Keywords:** cyclophosphamide, dexamethasone, diethylstilbestrol, T cell, thymus, toxicogenomics, 2,3,7,8-tetrachlorodibenzo-*p*-dioxin

## Abstract

**Background:**

Dysregulation of positive and negative selection, antigen presentation, or apoptosis in the thymus can lead to immunosuppression or autoimmunity. Diethylstilbestrol (DES), dexamethasone (DEX), cyclophosphamide (CPS), and 2,3,7,8-tetrachlorodibenzo-*p*-dioxin (TCDD) are immunosuppressive chemicals that induce similar immunotoxic effects in the thymus, however, the mechanism of toxicity is purported to be different for each compound.

**Objectives:**

We hypothesized that genomic analysis of thymus after chemical-induced atrophy would yield transcriptional profiles that suggest pathways of toxicity associated with reduced function.

**Methods:**

Female B6C3F1 mice were exposed to these immunosuppressive agents and changes in gene expression and immune cell subpopulations were evaluated.

**Results:**

All four chemicals induced thymic atrophy and changes in both the relative proportion and absolute number of CD3^+^, CD4^+^/CD8^−^, CD4^−^/CD8^+^, and CD4^+^/CD8^+^ thymocytes. The most significant impact of exposure to DEX, DES, and CPS was modulation of gene expression in the T-cell receptor (TCR) complex and TCR and CD28 signaling pathways; this could represent a common mechanism of action and play a pivotal role in lineage commitment and development of T cells. Up-regulation of genes associated with the antigen presentation and dendritic cell maturation pathways was the most distinctive effect of TCDD exposure. These elements, which were also up-regulated by DEX and DES, contribute to positive and negative selection.

**Conclusions:**

Genomic analysis revealed gene expression changes in several pathways that are commonly associated with xenobiotic-induced immune system perturbations, particularly those that contribute to the development and maturation of thymic T cells.

The immune system is a complex set of cellular, chemical, and soluble protein components that interact with each other in a sequential, regulated manner to protect the body against foreign substances. Early T-lymphocyte (T-cell) progenitors originate in the bone marrow and migrate to the thymus, where they differentiate, undergo positive and negative selection, and mature, before being released into circulation. Appropriate and carefully regulated apoptosis is one of the major mechanisms for homeostasis and the selection and development of fully competent T cells (Chaplin 2010). Recent efforts have sought to use transcriptional profiling to discover genomic patterns that are predictive of toxicity and to investigate the molecular responses to chemical stressors (Auerbach et al. 2010; Boverhof et al. 2009). Identifying chemical-induced changes in gene expression would contribute to a deeper understanding of the molecular mechanisms of immunotoxicity and would allow for an assessment of the immune system within the confines of traditional toxicology studies.

Exposure to the immunosuppressive agents dexamethasone (DEX) (Marchetti et al. 2003; van Vliet et al. 1986), diethylstilbestrol (DES) (Gould et al. 2000), cyclophosphamide (CPS) (Luster et al. 1981; Wang and Cai 1999), and 2,3,7,8-tetrachlorodibenzo-*p*-dioxin (TCDD) (Camacho et al. 2005; Dean et al. 1985) results in hypocellularity, atrophy, and apoptosis in the thymus; however, the putative chemical mechanisms of action and cellular targets differ (Luebke et al. 2006). The nitrogen mustard derivative CPS induces DNA mutation by alkylation and cross-linking (Wang and Cai 1999). DEX (Marchetti et al. 2003) and DES (Ahmed 2000), synthetic hormones that bind to the glucocorticoid and estrogen receptors, respectively, translocate to the nucleus bound to the receptors and regulate gene expression and protein synthesis. In the cytoplasm, DEX can bind to and interfere with regulatory factors, disrupt the T-cell receptor (TCR) complex, and impair T-cell signaling (Lowenberg et al. 2007). *In utero* DES exposure has been associated with postnatal alterations in T-cell and natural killer (NK) cell function and increased incidence of autoimmune diseases (Holladay 1999).

A potent and persistent environmental toxin, TCDD binds and activates the aryl hydrocarbon receptor (AhR) complex and, together with the AhR nuclear translocator, binds to dioxin-responsive elements in DNA to regulate gene expression and alter signal transduction pathways. TCDD diminishes both cell-mediated and humoral-mediated immunity and increases susceptibility to infectious disease and transplanted tumors (Luebke et al. 2006). The degree of immunosuppression induced, at a given dose, can be related to AhR binding affinity, but the mechanism by which this is achieved remains unconfirmed (Kerkvliet 2002; Luebke et al. 2006). It has been postulated that T-cell alteration occurs indirectly through other cells (Kerkvliet 2002), possibly during negative selection (Fisher et al. 2004) or by interaction with more TCDD-responsive cell types (Camacho et al. 2005; Kerkvliet 2002).

Based on the similarity of immunotoxic effects (i.e., thymus atrophy) induced by DES, DEX, CPS, and TCDD, we hypothesized that they would induce an overlapping transcriptional profile in the thymus reflective of general toxicity in this organ. In addition, because of differences in the reported mechanisms of action, we anticipated that each treatment would also lead to unique gene expression alterations indicative of perturbing specific molecular pathways. To test this hypothesis, we used an immune-focused array to evaluate transcriptional changes in pathways leading to altered immune status after treatment with DES, DEX, CPS, or TCDD and correlated genomic profiles with changes in thymic cell populations. Samples were collected under conditions demonstrated previously to induce thymic atrophy and immunosuppression (reviewed by Dean et al. 1985), when transcriptional analysis might show which pathways remained functional and which were compromised. Microarray analysis indentified 249 immune-relevant genes that were differentially expressed in thymus by the high dose of one or more of the four chemicals, compared with their respective control.

Initial analyses of this data set were described by Patterson and Germolec (2006). That report included an enumeration of alterations in gene expression, clustering of expression profiles, and a limited, low-stringency survey of gene ontology. In this article we describe changes in the relative proportion of T-cell subsets in the thymus by all four chemicals and the use of pathway mapping techniques to reveal transcription changes in several critical pathways associated with T-cell development and immune system perturbation. The correlations among genomic analysis, cellular pathology, and putative mechanisms of action demonstrate the utility of this approach as an adjunct to routine toxicity testing for hazard identification.

## Materials and Methods

### Animals and treatment

Female pathogen-free B6C3F1 mice were obtained from Taconic (Rockville, MD) at 4–8 weeks of age and maintained on a 12-hr light/dark cycle at 20–22°C in a facility accredited by the Association for Assessment and Accreditation of Laboratory Animal Care. All experiments were conducted under a protocol approved by the Virginia Commonwealth University Animal Care and Use Committee, and animals were treated humanely and with regard for alleviation of pain and distress. The animals received Harlan Teklad (Madison, WI) Laboratory Diet 7022 (NIH07) and water *ad libitum*. At 11–12 weeks of age, the mice were treated with one of four experimental chemicals, DES, DEX, CPS (all from Sigma-Aldrich Corp., St. Louis, MO), or 2,3,7,8-tetracholorodibenzo-*p*-dioxin (TCDD; Research Triangle Institute, Research Triangle Park, NC), at doses that have been demonstrated to induce thymic atrophy and immunosuppression, or a matched vehicle, for 5 days (Table 1). The route, dose, and vehicles for all of the chemicals used in this study were selected based on reviews included in Dean et al. (1982, 1985) and on work published by Luster et al. (1981) and van Vliet et al. (1986). On day 6, the mice were weighed and euthanized by carbon dioxide asphyxiation, and the thymus was excised and weighed. For microarray experiments, thymi (*n* = 4) were stored in RNAlater (Ambion, Austin, TX) at −20°C until RNA isolation.

### RNA isolation and amplification

Total RNA was extracted via the Qiagen RNeasy kit (Qiagen, Valencia, CA); evaluated by absorbance 260/280, gel electrophoresis, and Agilent 2100 Bioanalyzer analysis (Agilent Technologies, Palo Alto, CA); and amplified and purified using the Ambion Illumina RNA amplification kit according to the manufacturer’s instructions. Briefly, 400 ng of total RNA was reverse transcribed to cDNA, which was transcribed to cRNA and labeled with biotin-16-UTP. The cRNA was quantified using the RiboGreen RNA Quantitation Kit (Molecular Probes, Eugene, OR).

### Microarray

Labeled cRNA samples were hybridized to a custom Illumina Sentrix Array Matrix (Illumina, Inc., San Diego, CA) for 16–18 hr at 55°C, following the manufacturer’s instructions. The matrix contains oligonucleotide arrays, consisting of 698 genes selected to reflect responses relevant to immune function plus housekeeping genes [two 50-mer probes/gene; see Supplemental Material, Table 1 (doi:10.1289/ehp.1002358)] and 12 negative control sequences (710 genes total), arranged in a 96-well design. The arrays were washed, blocked with casein, incubated with streptavidin-Cy3, dried, and scanned on the Illumina BeadArray Reader GX.

### Microarray data analysis

Microarray data were analyzed using Illumina BeadStudio software and Ingenuity Pathway Analysis (IPA; version 8.0; Ingenuity Systems, Redwood City, CA). In BeadStudio, intensity data from the BeadArray Reader were normalized using a rank invariant algorithm with a linear scaling factor, determined by the rank-invariant genes. Differential expression (DIFF) of each probe was evaluated using an error model that calculates a *p*-value as a function of intensity differential and biological, technical, and nonspecific variation. A DIFF score of ± 20 corresponds to a *p*-value of 0.01; DIFF scores of corresponding probes were averaged, and concordance between probes was calculated. Detection scores calculated using negative control sequences as a reference were used to distinguish true expression from background noise. Only genes for which the absolute value of the DIFF score ≥ 20 and that had concordance and detection scores ≥ 0.95 were stratified by functional and pathway characterization in IPA. The Ingenuity Pathway Knowledge Base was used as the reference set. Fisher’s exact test was used to calculate a *p*-value determining the probability that the association between the genes in the data set and the function or pathway would be explained by chance alone.

### Flow cytometric analysis

Thymocytes (*n* = 8 mice) were isolated and cell surface markers quantified as previously described (Guo et al. 2006). Single-cell suspensions were incubated with appropriate monoclonal antibodies (BD Pharmingen, San Diego, CA) and propidium iodide (PI). Fluorescein isothiocyanate (FITC)–conjugated CD3, phycoerythrin-conjugated CD4, and FITC-conjugated CD8a were used to detect specific cell populations, which were counted on a Becton Dickinson FACScan Flow Cytometer (BD Biosciences Immunocytometry Systems, San Jose, CA). Nonviable cells and red blood cells (RBCs) were eliminated using a gate setting that excluded PI fluorescence and a forward scatter threshold above RBC size. For each sample, 5,000 PI-negative events were counted. Raw data were analyzed using CellQuest software (BD Biosciences Immunocytometry Systems). Homogeneity was determined using Bartlett’s test. Homogeneous data were evaluated using a parametric analysis of variance and Dunnett’s *t*-test. Nonhomogeneous data were evaluated using a nonparametric analysis of variance and the Wilcoxon rank sum test.

## Results

### General parameters of toxicity and alterations in thymic lymphocyte populations

**Figure 1:**
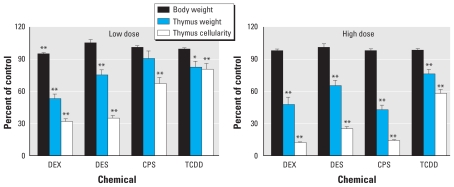
Three indicators of toxicity, body weight, thymus weight, and thymus cell number, measured to evaluate acute toxicity and thymic atrophy after low dose (*A*) or high dose (*B*) of immunosuppressive agents (percent control; mean ± SE, *n* = 8). *p ≤ 0.05; **p ≤ 0.01 versus matched control.

Consistent with published literature, all four chemicals induced thymic atrophy, as evidenced by significantly decreased organ weight and cellularity compared with the appropriate control (Figure 1). We observed a 5% decrease in body weight in the low-dose DEX group; otherwise, treatment did not affect body weight. At the high dose, all four chemicals induced an elevation in the percentage of CD3^+^, CD4^+^/CD8^−^, and CD4^−^/CD8^+^ cells in the thymus (Table 2). In contrast, at the high dose, all four chemicals decreased the total number of CD4^+^/CD8^−^ cells; DEX, CPS, and TCDD decreased the total number of CD3^+^ cells; and DEX and CPS decreased the total number of CD4^−^/CD8^+^ cells, reflecting overall thymic atrophy. The percentage and total numbers of CD4^+^/CD8^+^ cells were suppressed by DEX, DES, and CPS at both the high and low doses and by TCDD at the high dose. Low-dose TCDD also suppressed the total number of CD4^+^/CD8^+^ cells. Changes in the number and proportion of these populations suggest alterations in T-cell development and differentiation processes and in the availability of mature, functional T-helper and cytotoxic T lymphocytes (CTLs) for release into circulation. We also used two splenic T-cell functional assays to phenotypically anchor observed changes in gene expression with T-cell recognition and proliferative capability (Patterson et al. 2006). DES, DEX, and CPS, but not TCDD, suppressed T-cell proliferation in response to antibody directed against CD3 and to allogeneic leukocytes at 8.0, 5.0, and 50.0 mg/kg/day, respectively, and at 0.8 mg/kg/day DES, confirming the suppression of functional immune responses by these compounds under the conditions of this study.

### Global gene expression

Seventeen genes (*Ccnd3*↓, *Tcf7*↓, *Cdk2*↓, *Gfi1*↓, *Lat*↓, *Ifngr1*↓, *Capn2*↑, *Ifi16*↑, *Pbef1*↑, *Gpx3*↑, *Tyrobp*↑, *Klf2*↑, *Casp1*↑, *Fcer1g*↑, *Fcgr3*↑, *Apoe*↑, *Ly6a*↑) associated with T-cell development and differentiation, cellular homeostasis, and apoptosis, proliferation, and cell cycle regulation were differentially expressed (Illumina DIFF score, |≥ 20|; concordance and detection scores ≥ 0.95) by the high dose of all four chemicals. High-dose exposure to TCDD and CPS altered 73 and 98 genes, respectively; however, low-dose exposure altered very few genes (TCDD; 8 genes; CPS; 2 genes). Conversely, exposure to DEX and DES resulted in genes altered at the low dose only (DEX, 21 genes; DES, 21 genes), genes altered at both low and high doses (DEX, 149 genes; DES, 99 genes), and genes altered at the high dose only (DEX, 64 genes; DES, 46 genes). A complete list of all differentially expressed genes is included in Supplemental Material, Table 2 (doi:10.1289/ehp.1002358).

**Figure 2:**
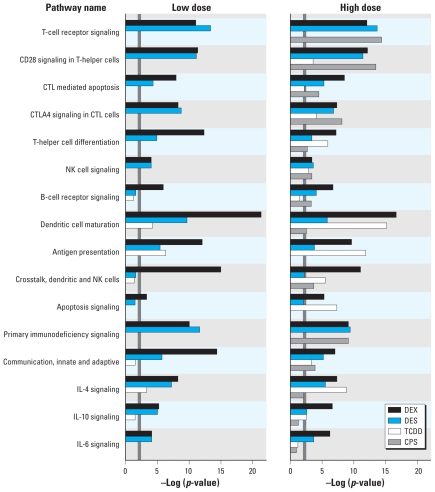
Global alterations in canonical pathways: significance scores (−log of *p*-value), calculated using Fisher’s exact test, for IPA pathways plotted for DES, DEX, CPS, and TCDD. The solid line at −log(*p*-value) of 2 represents a significance threshold at *p* = 0.01.

We mapped differentially expressed genes to pathways using IPA (for detailed lists, see Supplemental Material, Table 3A–C (doi:10.1289/ehp.1002358)]. The criteria for pathway inclusion were at least three differentially expressed genes and a *p*-value of ≤ 0.01 (Figure 2). The criteria for function inclusion were a *p*-value of ≤ 0.01 and a minimum of five genes for the global gene set or three genes for the common and unique gene sets [see Supplemental Material, Tables 3B,C and 4 (doi:10.1289/ehp.1002358)]. Because of the specific and directional nature of pathways, we chose them as the focus for our analyses. We gave more weight to pathways with strong trends in expression patterns (i.e., most genes similarly regulated), alterations in related steps, altered receptors, or groups of genes with broad-reaching effects and to pathways altered by multiple chemicals.

### T-cell–related pathways

At the high dose, DEX, DES, and CPS down-regulated genes in the TCR complex (*CD3d*, *CD3e*, *CD3g*) and profoundly affected three pathways anchored to the TCR: TCR signaling, CD28 signaling in T-helper cells, and CTL mediated apoptosis of target cells [Figure 2; see also Supplemental Material, Table 3A,B (doi:10.1289/ehp.1002358)]. TCDD exposure did not affect the TCR. Twelve genes in the TCR signaling pathway, including *CD3*, *CD4*, and *CD8*, were down-regulated by DEX (both doses), DES (both doses), and CPS (high dose); this group of genes also plays a key role in other T-cell–related pathways. The TCR signaling pathway scored the lowest *p*-value and included the largest number of genes altered in the DES and CPS gene sets. CD28 is a costimulatory protein for TCR signaling; CTLA-4 binds to the same molecules but transmits an inhibitory signal. Both pathways flow into the TCR signaling pathway. All four chemicals altered these pathways. DEX (both doses), DES (both doses), and CPS (high dose) down-regulated seven genes common to the TCR, CD28, and CTLA-4 pathways, including *CD28*, but no chemical altered *CTLA-4*. TCDD (high dose) and DEX (both doses) up-regulated an Fc-receptor gene associated with antigen binding and a small group of histocompatibility genes associated with antigen presentation. TCR genes were also a key component of the CTL-mediated apoptosis of target cells pathway, along with a group of apoptosis genes altered by DEX (high dose). The T-helper cell differentiation pathway included the Fc-receptor (↑, DEX, DES, CPS, TCDD), *CD28* (↓, DEX, DES, CPS), and histocompatibility (↑, DEX, TCDD) genes, but no other genes altered in the TCR or CD28 signaling pathways. Four cytokine receptors in the T-helper cell differentiation pathway were also altered by DEX. All four chemicals up-regulated receptor components (*Fcer1g*, *Tyrobp*) in the activation arm of the NK cell signaling pathway, and DEX, DES, and CPS down-regulated additional genes, including *Zap70*, a protein kinase that serves as a focal point for pathway signaling. All four chemicals altered function genes associated with T-cell proliferation and development, and DEX, DES, and CPS altered genes associated with T-cell activation and apoptosis [see Supplemental Material, Table 4 (doi:10.1289/ehp.1002358)].

### B-cell receptor signaling/B-cell functions

B- and T-cell signaling pathways have many molecules in common. Accordingly, the B-cell receptor signaling pathway was significantly altered by DEX (both doses), DES (both doses), and CPS (high dose) (Figure 2), with more than one-half of the altered genes, including receptor *B220/Ptprc*, down-regulated. DEX, DES, and CPS also altered several genes that affect the quantity of B cells produced.

### Antigen presentation and dendritic cell pathways

The most distinctive effects of TCDD exposure were up-regulation of the antigen presentation and dendritic cell (DC) maturation pathways. DEX and DES similarly altered these pathways, and CPS altered the DC maturation pathway (Figure 2). The most potent chemicals were DEX (both doses) and TCDD (high dose), up-regulating genes in the CD8^+^ and CD4^+^ arms of the antigen presentation pathway, major histocompatibility (MHC) I (*B2m*) and MHC II (*CD74*, *HLA-DMA*, *HLA-DMB*, *HLA*-*DQA1*, *HLA-DQB1*, *HLA-DRA*, *HLA-DRB1*) molecules, and three Fc receptors. At the high dose, all four chemicals also altered a related pathway, crosstalk between dendritic and NK cells, and general function categories related to quantity and chemotaxis of antigen-presenting cells (APCs); and DEX altered additional APC functions.

### Other pathways

We observed several changes in gene expression in interleukin (IL) and growth factor pathways [Figure 2; see also Supplemental Material, Table 3A (doi:10.1289/ehp.1002358)], most notably in receptors and in Janus kinases and signal transducers and activators of transcription molecules, which transmit signals from receptor to nucleus. DEX, primarily, and at least one other chemical altered receptor(s) in the IL-4, IL-10, IL-6, IL-15, interferon, transforming growth factor-β, and chemokine signaling pathways. DEX, DES, and CPS altered chemokine ligands, and DEX, DES, and TCDD altered several histocompatibility genes in the IL-4 pathway. Additionally, DEX altered an IL-2 receptor, and genes associated with the RelB arm of the nuclear factor κB signaling pathway. DEX (high dose) had the most profound effect on the apoptosis signaling pathway, altering nine genes, including *Fas*, *Bcl2*, and caspases 2, 3, and 6. TCDD and low-dose DEX also modulated this pathway, although to a smaller degree. Although DES and CPS did not significantly alter the apoptosis signaling pathway, they did modulate the function categories of T-cell proliferation and apoptosis. Pathways and functions affected by gene alterations common to three chemicals or unique to one chemical are presented in Supplemental Material [Table 3B,C (doi:10.1289/ehp.1002358)].

## Discussion

Exposure to environmental agents can compromise a number of immunological functions. In recent years, genomics has been used as an investigational tool to identify biomarkers and profiles indicative of disease and toxicity and to understand mechanisms of action (Auerbach et al. 2010; Boverhof et al. 2009). Future directions for toxicogenomics studies include coupling genomic data with systems biology and population exposure in full risk assessment studies, using surrogate tissues (blood) to establish concordance between target and nontarget tissues, and developing genomic screening strategies to predict toxicity before adverse pathology is evident (Cui and Paules 2010; Heinloth et al. 2004). Stremmel et al. (2005) and Heinloth et al. (2004, 2007) demonstrated that microarray, although generally less sensitive than reverse-transcriptase polymerase chain reaction but encompassing a significant portion of the genome, can detect subtle changes reflecting biological behavior in dysfunctional murine T cells and toxicant-exposed rat liver, respectively, when more traditional methods (flow cytometry, enzyme-linked immunosorbent assay, histology, and clinical chemistry) did not detect differences. We postulated that profiling genetic alterations in thymus after damage would help identify the major immune pathways that were compromised by chemical exposure, generate valuable information regarding potential mechanisms of action, and elucidate transcriptional profiles that could contribute to hazard identification.

DEX and DES induced similar gene expression patterns at both low and high doses. TCDD and CPS induced significant transcriptional changes primarily at the high dose. Although the magnitude of the changes induced by DEX and DES increased at the high dose, the same biological pathways, and key gene groups, were differentially expressed at both doses. Heinloth et al. (2004) have proposed that such a pattern attests to the sensitivity of microarray as a biologically relevant end point and indicator of potential adverse effects. DEX had the most significant and widespread effect on gene expression, although all treatments altered genes associated with T-cell proliferation, development, and apoptosis. This is consistent with the observed reductions in thymic weight and cellularity and the total numbers of all T-cell populations measured, as well as the selective and maturational events that occur in the thymus. Apoptosis is a critical process in the deletion of autoreactive T cells (Chaplin 2010). Individual chemicals regulated caspases 2 and 3, *Fas*, and members of the tumor necrosis factor receptor and B-cell lymphoma 2 families, focal points in death-receptor–mediated apoptosis. Many of the genes differentially expressed by all four treatments (high dose), specifically *Ccnd3* (Sasaki et al. 2007), *Fcer1g* (Shores et al. 1998), *Fcgr3* (Lynch 2000), *Gfi1* (Yucel et al. 2003), *Lat* (Chaplin 2010), *Ly6a* (Bamezai et al. 1995), and *Tcf7* (Willinger et al. 2006), have been associated with T-cell development and differentiation in the thymus. *ApoE* (Laskowitz et al. 2000), *Capn2* (Squier and Cohen 1997), *Casp1* (Zhou et al. 2000), *Cdk2* (Williams et al. 2000), *Gfi1* (Pargmann et al. 2007), *Ifi16* (Wei et al. 2003), *Ifngr1* (Matthys et al. 1995), and *Klf2* (Bai et al. 2007) contribute to apoptosis, proliferation, and cell cycle regulation, which are key components of negative selection. Collectively, these genes affect each stage of thymocyte development from the double-negative stage through release from the thymus into circulation. Dysregulation of these genes can lead to maturation arrest and inappropriate selection and apoptosis, and our findings support the utility of gene expression data to identify chemicals that may target the thymus.

The most significant effect induced by DEX, DES, and CPS was down-regulation of genes in the TCR complex and the TCR signaling and CD28 signaling pathways. This may represent a common mechanism for DEX, DES, and CPS and distinguish them from TCDD, which did not have an effect on the expression of the TCR complex or signaling. TCDD did alter the CD28 signaling pathway via histocompatibility genes. DEX, DES, and CPS down-regulated *CD3d, CD3e,* and *CD3g,* as well as the coreceptors *CD4*, *CD8a* and *CD8b*, and *CD28*. Several models have been proposed to track the evolution of thymic cells from CD4^+^/CD8^+^ through intermediate phenotypes to mature, functional T-helper cells and CTLs. One commonality among these models is the increase in TCR/CD3 expression at critical junctures in lineage commitment, followed by up- and down-regulation of the coreceptors CD4 and CD8 (Kydd et al. 1995; Marodon and Rocha 1994). High levels of CD3 are required (Kydd et al. 1995), and loss of the required molecules blocks developmental progression and results in thymocyte apoptosis (Killeen and Littman 1996). Mutation or suppression of kinases and other molecules has also been associated with signaling errors during selection processes that lead to impaired thymocyte development and alterations in CD4^+^ and CD8^+^ cell populations (Rudd et al. 2006). In our studies, each of the four chemicals tested induced suppression of both total number and percentage of CD4^+^/CD8^+^ precursor cells and the total number of CD4^+^/CD8^−^ cells. DEX and CPS exposure also led to a decrease in the number of CD4^−^/CD8^+^ cells, whereas the percentages of CD3^+^, CD4^+^/CD8^−^, and CD4^−^/CD8^+^ cells were increased by all chemicals. The degree to which any gene or set of genes controls the outcome of T-cell maturation has not been definitively established. However, it is possible that the down-regulation of *CD3*, *CD4*, *CD8*, and the TCR signaling pathway by DEX, DES, and CPS could impair T-cell development, leading to a loss of precursor cells by apoptosis and subsequent reduction in the number of mature T cells.

A minority cell population in the thymus, thymic B cells, found primarily in the medulla, are specifically adapted to the processes of negative selection and contribute to the repertoire of developing T cells (Spencer et al. 1992). DEX, DES, and CPS, but not TCDD, altered a common set of genes associated with the B-cell signaling pathway and B-cell proliferation and quantity, reflecting the interaction of B cells and T cells in functional and efficient thymic maturation.

The thymus houses a diverse collection of DCs and APCs that present MHC-bound self-antigens to developing T cells and induce negative selection and apoptosis in potentially autoreactive T cells (Evans et al. 2008). The expression patterns in the antigen presentation pathway were virtually identical for DEX and TCDD, suggesting a common mechanism of action. Both MHC I and MHC II genes were up-regulated. These distinct groups of genes, along with Fc receptor genes, were the major genes altered by TCDD and DEX in the DC maturation pathway. The reported effects of DEX on function and T-cell interaction with DCs vary with the conditions of exposure and type of DC studied (Butts et al. 2007; Matyszak et al. 2000). Modulation of pathways associated with antigen presentation was the most significant effect induced by TCDD in this study, whereas the T-cell signaling pathway was unaffected. Toxic insults to APC populations, and expression of MHC molecules in particular, can adversely affect T-cell function and activity. For example, TCDD altered the CD28 signaling pathway via MHC and Fc genes. TCDD has been shown to have direct effects on APC from several tissues, altering cell number, differentiation, activation, expression of MHC II, and the interaction of DCs with T cells (Lee et al. 2007; Vorderstrasse et al. 2003), and to alter expression of several genes involved in negative selection and apoptosis in the thymus (Fisher et al. 2004). It has been proposed that the inappropriate activation of cells, as is suggested both in *in vivo* studies and our gene expression data, may be the more significant mechanism for TCDD-induced immunotoxicity, rather than a direct suppression of immune functions (Kerkvliet 2002). This inappropriate activation of cells could lead to the thymic atrophy and immune system dysregulation that occurs after TCDD treatment, and may represent a novel mechanism of action for TCDD.

## Conclusions

The transcriptional profiles of DEX, DES, CPS, and TCDD identified a number of immune pathways and functions that were modulated after chemical exposure. Consistent with the thymic atrophy and changes in thymic lymphocyte populations, the constellation of pathways affected suggests dysregulation of T-cell development in the thymus and function of mature T cells upon release. Although only DEX and TCDD had a significant impact on the apoptosis signaling pathway, each of the four chemicals altered genes associated with apoptosis function, a critical factor in T-cell selection. The most significant observations were the strong down-regulation of the TCR complex and the TCR and CD28 signaling pathways by DEX, DES, and CPS and up-regulation of the antigen presentation and DC maturation pathways by TCDD, in particular, and by DEX and DES. Consistent with published research, these findings suggest that DEX, DES, and CPS act primarily through direct action on developing T cells in the thymus, whereas TCDD acts via an alternate mechanism. The full mechanism of action of a chemical is multifactorial and dependent upon the tissue and conditions of exposure. However, genomic analysis can be a useful tool in evaluating the most significant immune system perturbations induced and identifying those chemicals that may have potential to affect human health through alteration of functional immunity.

## Figures and Tables

**Table 1:** Animals and treatment.

	Chemical			
Treatment	DES	DEX	CPS	TCDD
High dose	8.0 mg/kg/day	5.0 mg/kg/day	50.0 mg/kg/day	3.0 μg/kg/day
Low dose	0.8 mg/kg/day	0.5 mg/kg/day	5.0 mg/kg/day	0.3 μg/kg/day
Vehicle	Corn oil	Ethanol/saline	Phosphate-buffered saline	Corn oil
Route of exposure	Subcutaneous injection	Intraperitoneal injection	Intraperitoneal injection	Gavage

Female B6C3F1 mice 11 to 12 weeks of age were used for microarray (*n* = 4) and immune assays (*n* = 8). For all treatments, mice were dosed once daily for 5 consecutive days at the specified concentration.

**Table 2:** Lymphoid cell populations in the thymus (mean ± SE, *n* = 8).

	Percent total cells^a^				Cells/thymus × 10^6^^b^			
Exposure	CD3^+^	CD4^+^/CD8^−^	CD4^−^/CD8^+^	CD4^+^/CD8^+^	CD3^+^	CD4^+^/CD8^−^	CD4^−^/CD8^+^	CD4^+^/CD8^+^
DEX								

Vehicle	14.2 ± 0.5	11.9 ± 0.2	2.7 ± 0.1	78.9 ± 0.2	23.6 ± 1.3	19.8 ± 1.1	4.5 ± 0.3	131.3 ± 0.6
0.5 mg/kg/day	37.0 ± 1.5**	29.3 ± 1.0**	8.4 ± 0.4**	34.6 ± 2.4**	18.9 ± 1.6*	14.9 ± 1.2**	4.3 ± 1.4	18.2 ± 2.7**
5 mg/kg/day	31.4 ± 2.2**	19.7 ± 2.3**	8.0 ± 1.0**	0.5 ± 0.2**	6.2 ± 0.7**	4.0 ± 0.7**	1.6 ± 0.3**	0.1 ± 0.1**

DES								

Vehicle	13.5 ± 0.3	12.7 ± 0.2	2.4 ± 0.1	79.7 ± 0.3	21.2 ± 1.2	20.1 ± 1.2	3.9 ± 0.3	126.0 ± 7.8
0.8 mg/kg/day	35.9 ± 1.6**	31.1 ± 1.2**	7.6 ± 0.5**	51.1 ± 2.2**	19.3 ± 0.8	16.7 ± 0.8	4.0 ± 0.2	28.6 ± 3.4**
8 mg/kg/day	50.9 ± 2.9**	42.3 ± 2.2**	10.6 ± 0.7**	32.9 ± 3.4**	19.5 ± 1.3	16.3 ± 1.2*	4.0 ± 0.3	13.5 ± 2.2**

CPS								

Vehicle	14.0 ± 0.6	11.4 ± 0.5	2.6 ± 0.1	78.3 ± 0.9	18.6 ± 1.5	15.2 ± 1.1	3.5 ± 0.3	105.7 ± 10.6
5 mg/kg/day	15.4 ± 0.5	12.5 ± 0.3*	3.2 ± 0.2*	73.7 ± 0.7**	13.9 ± 2.2*	11.2 ± 0.9*	2.8 ± 0.2	66.4 ± 5.9**
50 mg/kg/day	65.6 ± 4.0**	44.6 ± 3.1**	13.1 ± 1.2**	4.4 ± 2.3**	12.3 ± 1.1**	8.3 ± 0.8**	2.4 ± 0.3*	0.7 ± 0.4**

TCDD								

Vehicle	15.2 ± 0.2	12.6 ± 0.3	3.0 ± 0.1	78.1 ± 0.6	23.1 ± 0.5	19.1 ± 0.4	4.6 ± 0.2	118.7 ± 2.4
0.3 μg/kg/day	14.9 ± 0.3	11.7 ± 0.3	3.0 ± 0.1	79.1 ± 0.5	18.3 ± 1.4*	14.4 ± 1.2**	3.6 ± 0.3	96.5 ± 6.6**
3 μg/kg/day	19.7 ± 0.7**	14.7 ± 0.8*	4.6 ± 0.2**	72.0 ± 1.3**	17.2 ± 1.1**	12.8 ± 0.9**	4.0 ± 0.3	63.4 ± 4.6**

a Values represent percentage gated lymphocyte population using CellQuest software.

b Percent values and cell counts were used to calculate the total number of each cell population in the thymus.

* *p* ≤ 0.05

** *p* ≤ 0.01, versus matched control.
